# The Effects of Diet on the Proportion of Intramuscular Fat in Human Muscle: A Systematic Review and Meta-analysis

**DOI:** 10.3389/fnut.2018.00007

**Published:** 2018-02-20

**Authors:** Sara Ahmed, Dhanveer Singh, Shereen Khattab, Jessica Babineau, Dinesh Kumbhare

**Affiliations:** ^1^McMaster University, Hamilton, ON, Canada; ^2^Royal College of Physicians and Surgeons in Ireland, Dublin, Ireland; ^3^Library and Information Services, Toronto Rehabilitation Institute, University Health Network, Toronto, ON, Canada; ^4^Division of Physical Medicine and Rehabilitation, Department of Medicine, University of Toronto, Toronto, ON, Canada

**Keywords:** intramuscular fat, diet, review, high-fat diets, energy

## Abstract

**Background:**

There is an increasing trend in the consumption of poor-quality diets worldwide, contributing to the increase of non-communicable diseases. Diet directly influences physiological composition and subsequently physical health. Studies have shown that dietary macronutrient and energy content can influence the proportion of intramuscular fat (IMF), which mediates various metabolic and endocrine dysfunction. The purpose of this systematic review was to identify evidence in the literature assessing the association between different dietary interventions on the proportion of IMF in humans.

**Methods:**

Three medical databases were investigated (Medline, EMBASE, and Cochrane) to identify studies assessing changes in IMF after dietary interventions. The primary outcome measure was the change in IMF proportions after a dietary intervention. The effects of high-fat, high-carbohydrate, low-calorie, and starvation diets were assessed qualitatively. A meta-analysis assessing the effect of high-fat diets was conducted. Follow-up sensitivity and subgroup analyses were also conducted.

**Results:**

One thousand eight hundred and sixty-six articles were identified for review. Of these articles, 13 were eligible for inclusion after a full screening. High-fat diets increased IMF proportions, standardized mean difference = 1.24 (95% confidence interval, 0.43–2.05) and a significant overall effect size (*P* = 0.003). Diets with an increased proportion of carbohydrates decreased IMF proportions; however, increasing caloric intake with carbohydrates increased IMF. Starvation diets increased IMF stores, and hypocaloric diets did not result in any IMF proportion changes.

**Conclusion:**

This systematic review suggests that high-fat diets and diets with caloric intake increased above the amount required to maintain BMI with carbohydrates, and short-term starvation diets are associated with increases in IMF content. Further studies are needed to assess the effects of macronutrient combinations on IMF and the influence of diet-induced IMF alterations on health outcomes. In addition, IMF poses a possibly effective clinical marker of health.

## Introduction

The prevalence of poor-quality diets has increased worldwide in the past two decades, presenting as a main contributor to the increasing rates of chronic illness and mortality (1, 2). A study by Ward et al. (3) showed that one in four adults had two or more chronic health conditions. This is projected to increase in the coming years, accounting for 62% of worldwide deaths (4–6). Increases in body fat deposition increase the risk of developing non-communicable diseases. Intramuscular fat (IMF) proportions are principal mediators of various metabolic and endocrine functions that lead to these diseases (7, 8).

Studies have shown that dietary macronutrient distribution and energy content can influence the proportion of IMF (9–11). Common diet interventions include various combinations of fat, carbohydrate, and protein proportions as well as low-calorie and starvation diets. Increases in fat consumption, saturated or unsaturated, has been correlated with increases in the proportion of IMF in rodents, cattle, and porcine (12, 13). Buettner et al. (14) and van den Broek et al. (15) report increases in the proportion of IMF with high-fat diets in rodents. Similarly, high-fat diets are also associated with increases in IMF levels in humans (16). The effects of high-carbohydrate and low-calorie/starvation diets on IMF present conflicting results in the literature. Lapachet et al. (17) report unchanging IMF content in rats following a high-carbohydrate diet. In humans, Kiens et al. (9) and Maersk et al. (11) reported increases in IMF with increased dietary intake of carbohydrates. Very low-calorie or starvation diet interventions result in conflicting IMF content changes. Starvation diets induce IMF loss in porcine (18). In humans, there are reports of significantly higher proportions of IMF following starvation or low-calorie interventions (19, 20). Conversely, Larson-Meyer et al. (10) reported no significant changes in IMF with a low-calorie diet intervention compared to a control diet. It should be noted that changes in the proportion and metabolism of IMF can depend upon physical fitness. For example, in the so-called athlete paradox, high IMF levels are present in highly trained endurance athletes as a result of physiological adaptations to training (21); however, the influence of exercise training on IMF regulation is beyond the scope of this review. Since IMF mediates metabolic and endocrine functions, assessing the influence of diet on IMF levels is of clinical importance.

Increases in IMF have been implicated in the development of negative health outcomes such as metabolic syndrome and poor muscle strength, presenting a risk for the progression of chronic illness (22, 23). With the increase of nutrient-poor and energy-dense diets, predominantly the result of increases in high-fat and high-carbohydrate foods, the impact of dietary composition on IMF is important to elucidate (1). Poor IMF sequestering and higher levels of stored IMF have been associated with diseases such as diabetes and obesity and were found to mediate physiological functions such as insulin sensitivity (23–25). For instance, higher IMF levels are associated with increased insulin resistance. IMF may serve as a clinical marker of health status and diseases progression, as mounting evidence suggests that IMF is a significant mediator of chronic illness and an indicator of musculoskeletal health. IMF proportions can be readily imaged clinically using ultrasound and magnetic resonance imaging modalities (26–29). Currently, there are no systematic reviews assessing the effect of diet on IMF in humans. The purpose of this systematic review is to identify and evaluate literature assessing the association between different dietary interventions on the proportion of IMF in humans. The results of this study will provide insight into the anatomical changes associated with dietary intake and may provide useful implications for clinical dietary recommendations.

## Materials and Methods

This study was performed in accordance with the Preferred Reporting Items for Systematic Reviews and Meta-analyses (PRISMA). An information specialist conducted a comprehensive systematic search of the literature in Medline (including Medline ePub Ahead of Print, In Process, and Other Non-Indexed Citations), EMBASE, and the Cochrane CENTRAL Register of Controlled Trials. Searches in each database were conducted from inception of the database to January 2017. The search strategies broadly searched for text words describing the words IMF, lipids triacylglycerol, or triglyceride. Searches were then limited to English papers and human populations. Google scholar was also searched to identify any missed articles. The full Medline search can be viewed in Appendix A.

Randomized controlled trials (RCTs), quasi-RCTs, controlled trials, randomized controlled crossover trials, or controlled crossover trials were included in this study. Crossover studies were included as they present low between-group variability and lend robust findings, particularly in studies with multiple dietary interventions. Systematic reviews, case–control, and cohort studies were excluded. Studies assessing the effects of interventions on pediatric populations, populations with chronic illness, and animal studies were excluded from this review because our purpose is to investigate dietary effects on humans with mature physiology. We included studies assessing the effects of any dietary intervention that implemented macronutrient modifications. Our primary outcome was the change in IMF proportions after a dietary intervention. Studies presenting changes associated with IMF physiology were excluded, as we were interested in the outcome of dietary intervention on IMF proportions rather than the mechanism to achieve these anatomical changes.

Two reviewers, SA and DS, independently assessed the titles and abstracts eligible for a full screen, and conflicts were resolved by a third reviewer, SK. Reviewers SA and DS screened full articles and determined those to be included in the review. Reviewers SA and DS completed data extraction independently and risk of bias assessments without blinding to authorship or journal. The risk of bias in the articles was determined using the checklist proposed by Downs and Black (30) for methodological quality in healthcare intervention studies. The criteria assessed were selection, performance, measurement, attrition, and reporting.

### Data Analysis

Studies were assessed qualitatively for the sample size, study design, proportion of men and women, age of the sample population, the macronutrient distributions or lack thereof in low/calorie starvation diets, the muscle assessment site, and the measurement modality. Results from each study were summarized, and trends associated with changes in IMF after different diet interventions were described.

The effect measures chosen were standardized mean differences (SMDs) for continuous data. Uncertainty was expressed using 95% confidence intervals (CIs). A meta-analysis was conducted to estimate pooled SMDs for each category using the RevMan© software (version 5.3.5, Software RevMan, Cochrane Collaboration). A random-effects model (DerSimonian and Laird method) was used to estimate the pooled SMD due to the variance in study interventions. The inverse variance method was used to calculate the pooled SMD. If a study presented multiple intervention comparisons or body area for muscle biopsy or scan, this was reported as a separate entry in the meta-analysis. Cohen’s criteria were used to determine the effect size of SMDs, where a SMD between 0.2 and 0.5 is small, and a SMD between 0.5 and 0.8 is moderate, and a SMD above 0.8 is large (31). SMDs below 0.2 were considered unsubstantial.

The χ^2^ test was used to assess heterogeneity with an alpha of 0.05. The *I*^2^ test for heterogeneity was used, with <25% representing low heterogeneity, 25–50% representing moderate heterogeneity, and over 50% representing high heterogeneity (32). Visual inspection of a funnel plot and an Egger’s test for funnel plot asymmetry was planned to assess for publication bias; however, there were too few studies (<10) in each intervention group to substantiate a powerful study. Clinical and methodological variability was assessed qualitatively to determine the sources of heterogeneity. Clinical variables may include age, physical activity, and gender of study participants. Methodological variables may include duration of dietary intervention, type characteristics of the dietary intervention, and blinding of assessors. A subgroup analysis was conducted to quantitatively ascertain sources of heterogeneity. Percent males, physical activity, trial type, assessed muscle, and measurement technique were included as subgroups. A sensitivity analysis was also conducted by removing studies with a minimum group sample size below 8, those who did not elucidate participant characteristics (gender, age, physical activity), and those with a higher risk of bias.

## Results

**Figure 1:**
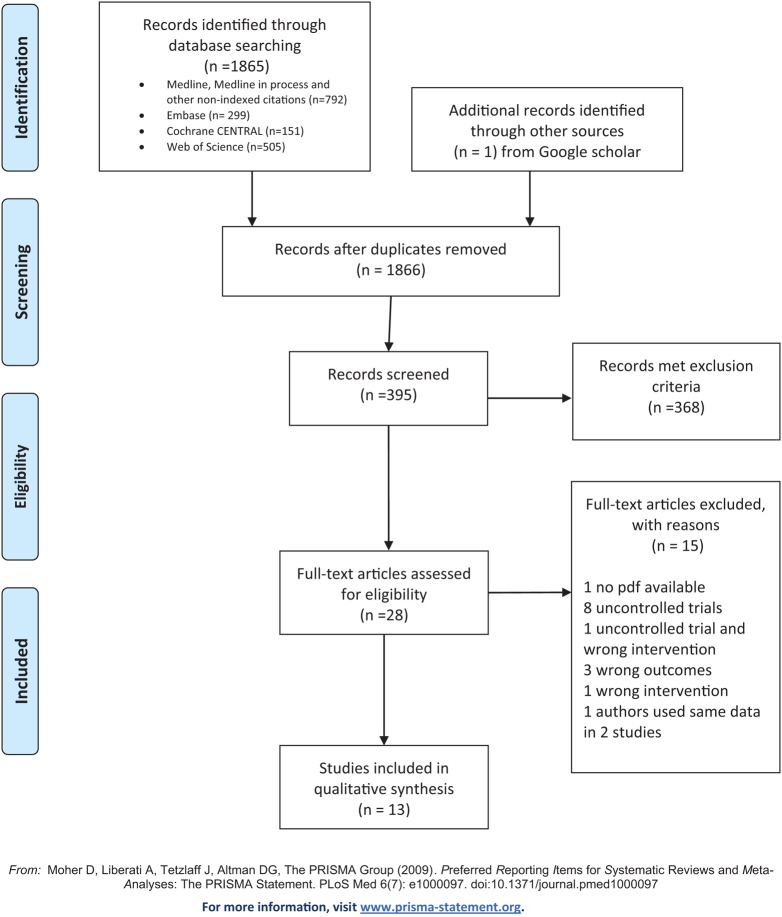
Preferred Reporting Items for Systematic Reviews and Meta-Analysis flow diagram.

After the removal of duplicates, 1,866 studies were identified for preliminary screening. The title and abstract screening process identified 28 potential articles for full screening. After screening the full articles (13 articles) remained for qualitative and quantitative synthesis (see Figure 1 for a PRISMA diagram).

The risk of bias in the screened articles was low. Specific significance levels, random variability measures, description of sample characteristics, and attrition with the characteristics of removed participants were described in these studies. The study by St-Onge et al. (33) presented large attrition, as they reported a decrease in participants from 45 to 33 and then IMF measurements from 24 of the 33 participants. External validity and compliance was achieved in most studies. These studies applied stringent protocols to ensure participants’ adherence to the dietary interventions. Regular follow-ups, educational sessions, and diary recordings of diet were also consistent among all of the articles. All studies recruited participants through convenience sampling. Physical activity was well controlled for in all the studies. Sedentary participants were prohibited from physical activity. Activity remained constant among active participants groups, reducing the probability for exercise to present as a confounding variable. All studies consistently measured IMF proportions from lower body muscles, which included the vastus lateralis, tibialis anterior, and the soleus muscles. Eight articles took measurements from the vastus lateralis muscle. Three studies assessed the soleus muscle, and two assessed the tibialis anterior muscle. Of all the studies reviewed, only two studies specified blinding of data assessors and/or participants (19, 34). A detailed report on the risk of bias in the included studies can be found in Table 1.

**Table 1:** Quality assessment of articles.

	Hypothesis/aim/objective clearly described?	Main outcomes clearly described in the Introduction/Methods?	Characteristics of patients clearly described?	Interventions of interest clearly described?	Distribution of principal confounders in each group of subjects to be compared clearly described?	Main findings of the study clearly described?	Does the study provide estimates of random variability in the data for main outcomes?	Have all important adverse events that may be a consequence of intervention been reported?	Have the characteristics of patients lost to follow-up been described?	Have actual probability values been reported for main outcomes except where probability is <0.001?	Were the subjects asked to participate in the study representative of the entire population from which they were recruited?	Were those subjects who were prepared to participate representative of the entire population from which they were recruited?	Were the staff, places, and facilities where the patients were treated, representative of the treatment the majority of patients receive?	Was an attempted made to blind study subjects to the intervention they received?	Was an attempt made to blind those measuring the main outcomes of the intervention?	If any results of were based on “data dragging,” was it made clear?	In trials/cohort studies, do the analyses adjust for different lengths of follow-up, or in case–control studies, is the time period between intervention and outcome the same for cases and controls?	Were the statistical tests used to assess main outcomes appropriate?	Was compliance with the intervention(s) reliable?	Were the main outcome measures used accurate (valid and reliable)?	Were the patients in different intervention groups (trials and cohort studies) or were the cases and controls (case–control studies) recruited from the same population?	Were study subjects in different intervention groups (trials and cohort studies) or were the cases and controls (case–control studies) recruited over the same period of time?	Were study subjects randomized to intervention groups?	Was the randomized intervention assignment concealed from both patients/staff until recruitment was complete and irrevocable?	Was the adequate adjustment for confounding in the analyses from which the main findings were drawn?	Were losses of patients to follow-up taken into account?	Did the study have sufficient power to detect clinically important effect where*P*value for difference being due to chance is <5%?	Total score (31)

(20)	Y	Y	Y	Y	Y	Y	Y	N	Y	Y	N	N	N	Y	N	Y	Y	Y	Y	Y	Y	Y	N	N	Y	Y	4	23
(10)	Y	Y	Y	Y	N	Y	Y	N	Y	N	Y	Y	Y	Y	N	Y	Y	Y	Y	Y	Y	Y	N	N	Y	Y	5	25
(39)	Y	Y	Y	Y	Y	Y	Y	N	Y	N	N	N	Y	N	N	Y	Y	Y	N	Y	Y	Y	N	N	Y	Y	3	20
(37)	Y	Y	Y	Y	Y	Y	Y	N	Y	N	N	N	Y	N	N	Y	Y	Y	Y	Y	Y	N	Y	Y	N	Y	5	23
(33)	Y	Y	Y	Y	Y	Y	Y	N	Y	Y	N	N	Y	N	N	Y	Y	Y	N	Y	N	N	Y	N	Y	N	5	21
(38)	Y	Y	Y	Y	N	Y	Y	N	Y	Y	N	N	Y	N	N	Y	Y	Y	Y	Y	N	N	Y	N	N	N	5	20
(34)	Y	Y	Y	Y	Y	Y	Y	N	Y	N	N	N	Y	Y	Y	N	Y	Y	Y	Y	N	N	Y	Y	N	N	5	22
(9)	Y	Y	Y	Y	N	Y	Y	N	N	N	N	N	Y	N	N	Y	Y	Y	Y	Y	Y	N	N	N	N	N	5	19
(35)	Y	Y	Y	Y	N	Y	Y	N	Y	Y	N	N	N	N	N	Y	Y	Y	Y	Y	N	N	N	N	Y	Y	5	20
(36)	Y	Y	Y	Y	N	Y	Y	N	Y	Y	N	N	N	N	N	Y	Y	Y	Y	Y	Y	N	N	N	N	Y	5	20
(19)	Y	Y	Y	Y	Y	Y	Y	N	Y	Y	Y	N	N	N	Y	Y	Y	Y	Y	Y	N	N	Y	N	N	Y	3	21
(11)	Y	Y	Y	Y	Y	Y	Y	Y	Y	Y	Y	Y	Y	N	N	Y	Y	Y	Y	Y	Y	Y	Y	N	Y	Y	5	28
(40)	Y	Y	Y	Y	Y	Y	Y	N	Y	N	N	N	Y	N	N	Y	Y	Y	Y	Y	N	N	Y	N	N	Y	5	21

The dietary interventions in the 13 studies included high-fat diets, high-carbohydrate diets, normal diets with added carbohydrate consumption, low-calorie/starvation diets, and one intervention assessing the effect of a high-protein diet.

### Qualitative and Quantitative Analysis

Summaries of the included articles can be found in Table 2. Articles that provided absolute proportions of IMF were included in the meta-analysis. Studies that reported measurements using an IMF: water ratio or difference scores were not included in the meta-analysis. These studies were excluded from the meta-analysis because their metrics were not consistent with the remainder of the studies, and thus, a comparison could not be made. Methods used to quantify the amount of IMF in these studies were the following: magnetic resonance spectroscopy or muscle biopsy and chemical extraction, spectrophotometry, transmission electron microscopy, or oil red stain.

**Table 2:** Summary of findings.

Author	Sample size (N)	Age^a^	Study design	Sample characteristics	Intervention	Assessment site	Assessment method	Postintervention/IMF proportion
Larson-Meyer et al. (10)	M = 20, F = 26 (*N* = 46)	25–50	Randomized controlled trial	Healthy, sedentary overweight men and women	1. Control (C)	Soleus muscle	H^1^ magnetic resonance spectroscopy	No difference 25% calorie restriction and C/no difference between low calorie and C
					2. 25% calorie restriction of baseline energy requirements			
					3. Low-calorie diet until 15% reduction in weight			

Johnson et al. (20, 39)	M = 7 (*N* = 7)	30 (6)	Randomized crossover design	Healthy, physically fit males	1. Control: mixed carbohydrate diet (C)	Vastus lateralis	H^1^ magnetic resonance spectroscopy	HF > C, S > C/no difference HF and S
					2. Water-only starvation (S)			
					3. Low-carbohydrate/high-fat intake (HF)			

Green et al. (19)	M = 66 (*N* = 6)	38.8 (12.7)	Randomized crossover design	Healthy, physically fit men	1. Control: mixed diet (C)	Vastus lateralis	H^1^ magnetic resonance spectroscopy	S > C, S > HP/no difference HP and C
					2. Water-only starvation (S)			
					3. Low-carbohydrate/high-protein intake (HP)			

Johnson et al. (20, 39)	M = 6 (*N* = 6)	32 (2.2)	Randomized crossover design	Healthy, physically fit males	1. Control: high-carbohydrate diet (C)	Vastus lateralis	H^1^ magnetic resonance spectroscopy	HF > C
					2. Low-carbohydrate/high-fat diet (HF)			

St-Onge et al.. (33)	(*N* = 24)	44 (2.5)	Randomized crossover design	Healthy men and women with mildly elevated LDL	1. Control: low-fat diet (C)	Soleus	H^1^ magnetic resonance spectroscopy	HF > C
					2. High-fat diet (HF)			

Kiens et al. (9)	M = 19 (*N* = 19)	36 (30–40)	Controlled trial	Healthy, physically active males	1. Control diet (C)	Vastus lateralis	Biopsy and chemical extraction	HF > C/no difference HC and C
					2. High-fat diet (HF)			
					3. High-carbohydrate diet (HC)			

Sakurai et al. (35)	M = 37 (*N* = 37)	23.6 (0.5)	Randomized crossover design	Healthy, non-obese male volunteers	1. Control: normal fat diet (C)	Soleus and tibialis anterior	H^1^ magnetic resonance spectroscopy	i. HF > C, ii. HF > C
					2. High-fat diet (HF) i = soleus; ii = tibialis anterior			

Schrauwen-Hinderling (36)	M = 10 (*N* = 10)	25 (6.2)	Randomized crossover design	Healthy, young male subjects	1. Control: normal fat diet (C)	Vastus lateralis	H^1^ magnetic resonance imaging	No difference
					2. High-fat diet (HF)			

Skovbro et al. (37)	M = 21 (*N* = 21)	23.7 (2.74)	Randomized controlled trial	Healthy, untrained male subjects	1. Control: normal fat diet (C)	Vastus lateralis	Biopsy and spectrophotometry	No differences
					2. High-fat diet (HF)			

Sock et al. (40)	M = 11 (*N* = 11)	25 (0.6)	Randomized crossover design	Healthy, non-smoking males	1. Control diet (C)	Not specified	H^1^ magnetic resonance spectroscopy	HGlu > C/no difference HFru and C
					2. High-glucose diet (HGlu)			
					3. High-fructose diet (HFlu)			

Larson-Meyer et al. (34)	(*N* = 18)	18–45	Randomized crossover design	Healthy, endurance trained runners	1. Control: moderate fat diet (C)	Vastus lateralis	Biopsy and transmission electron microscopy	HC < C
					2. High-carbohydrate diet (HC)			

van Herpen et al. (38)	(*N* = 20)	55.2 (7.6)	Randomized controlled trial	Healthy, sedentary men	1. Control: low-fat diet (C)	Vastus lateralis	Biopsy and Oil Red stain	No difference between change C change vs. HF change
					2. High-fat diet (HF)			

Maersk et al. (11)	M = 17, F = 30 (*N* = 47)	20–50	Randomized controlled trial	Healthy, overweight, non-diabetic subjects	1. Control: water (C)	Tibialis anterior	H^1^ magnetic resonance spectroscopy	Sucrose/fructose difference > control/no difference milk and control
					2. 1 L of sucrose and fructose			
					3. Semi-skim milk			

*^a^Age is reported in mean (SD), mean, or range based on reporting in article*.

### High-Fat Diets

Eight studies investigated the effects of a high-fat diet on the proportion of IMF. Intervention groups consumed diets with a proportion of fat between 38 and 85%. Three studies instructed participants to consume 60% of their caloric intake in the form of fat, the remainder of the studies varied in the proportion of fat given to study participants (33–38). van Herpen et al. (38) and St-Onge et al. (33) instructed participants to consume 55 and 38% of their caloric intake in the form of fat. Carbohydrate levels were varied to accommodate for the increase in fat proportion. Protein intake was consistent among studies, constituting 15% of the experimental and control diets. Only one study assigned 20% protein as a component of the experimental and control diet (35).

Four of the six studies assessing the vastus lateralis muscle found a significant increase of IMF after a high-fat diet intervention (9, 20, 35, 39). These studies implemented a 54–83% fat diet in their experimental conditions. Schrauwen-Hinderling et al. (36) and Skovbro et al. (37) did not find any significant changes in the proportion of IMF within the vastus lateralis muscle after a high-fat (55–60% fat) diet. van Herpen et al. (38) found significant changes in IMF from baseline within the high-fat (55% of diet fat) and the control diet (25% fat in diet) group; however, they did not find any between-group differences after the interventions. Studies assessing changes in IMF in the soleus muscle found a significant increase in IMF after a high-fat diet that consisted of 38% fat relative to 30% fat diet and a 60% fat relative to a 25% fat diet (33, 35). The study assessing IMF changes in the tibialis anterior muscle also found a significant increase in IMF in the high-fat diet group (60% fat diet) relative to the control group (35). There was a 30% increase in the IMF in the tibialis anterior muscle of the high-fat diet group relative to the control group, and a 20% increase was found in the soleus muscle of the high-fat diet group relative to the control group. The studies by Johnson et al. (20) and Sakurai et al. (35) reported the largest proportion difference of IMF following a high-fat diet relative to a control diet. Skovbro et al. (37) reported the smallest proportion difference.

### High-Carbohydrate Diets

Four studies assessed the effect of increased carbohydrate intake on IMF (9, 11, 34, 40). Two studies increased the experimental groups’ carbohydrate intake and reduced their fat intake to compensate for this proportion change (9, 34). Larson-Meyer et al. (34) instructed participants to consume 75% of their daily caloric intake in the form of carbohydrates and 10% in the form of fat, relative to 50% carbohydrates and 35% fat in the control group. Protein intake was maintained at 15% of participants’ daily caloric intake. Kiens et al. (9) fed the high-carbohydrate group 51 and 38% of their daily caloric intake in the form of carbohydrates and fat, respectively, relative to 42 and 43% in the control group. Protein consumption remained constant at 15%. The remaining two studies added excess carbohydrates to their participants’ diets. Sock et al. (40) had two experimental groups, one that consumed 35% more energy in the form of glucose and the other consumed 35% more energy in the form of fructose. All participants, including the control group, consumed a standardized diet that consisted of 55% carbohydrates, 30% fat, and 15% protein. Maersk et al.’s (11) study participants received 1 L of a sucrose fructose drink (106 g of added carbohydrates), 1 L of semi-skim milk (41 g of added carbohydrates), or the control diet that was to maintain the composition of their current diet.

Two studies assessed the proportion of IMF in the vastus lateralis muscle, with one study utilizing chemical extraction and staining and the other using transmission electron microscopy to perform their measurements (9, 34). One study assessed IMF in the tibialis anterior muscle using H^1^MRS (11). One study reported using H^1^MRS as their measurement tool, but they did not specify the muscle they assessed (40). The studies assessing the impact of increased dietary carbohydrate-to-fat ratio observed decreases in IMF content of the vastus lateralis muscle in the high-carbohydrate diet group relative to the control diet (9, 34). Studies assessing the addition of carbohydrate sources to a normal diet found significant increases in the proportion of IMF in the assessed muscles (11, 40). Sock et al. (40) found that a high-fructose diet increased IMF proportions more than a high-glucose diet. Maersk et al. (11) found a 221% increase in IMF proportions after consuming 1 L of glucose fructose solution (106 g of carbohydrates) for 6 months relative to people consuming 1 L of water as a control. In addition, consuming semi-skim milk (added 41 g of carbohydrates) resulted in a 25% decrease in IMF proportions relative to consuming water.

### Low-Calorie and Starvation Diets

Three studies assessed the effects of low-calorie and starvation diets on the proportions of IMF (10, 19, 20). Larson-Meyer et al. (10) compared the effects of a 25% calorie reduced diet from weight maintenance energy requirements and a very low-calorie diet (890 cal per day) until 15% weight reduction, with control subjects on a weight maintenance diet. Participants were exposed to these interventions for 8 days with a 3-week washout period in between. Johnson et al. (20) and Green et al. (19) studied the influence of short-term starvation diets relative to control weight maintenance diets on IMF. Johnson et al. (20) and Green et al. (19) exposed participants in the starvation group to 67 h of water-only starvation, after 65 h of starvation measurements of IMF were taken. There was no change in IMF concentrations following a 25% calorie reduction diet and a very low-calorie diet relative to control diets (10). Starvation diets resulted in significantly higher IMF proportions relative to control diets (19, 20). These studies were composed of physically active men.

### High-Protein Diet

Green et al. (19) (*n* = 6), assessed whether a high-protein low-carbohydrate diet would influence IMF proportions. Participants in the experimental group consumed diets consisting of 35% fat and 65% protein, with a negligible consumption of carbohydrates. The control group consumed 50% of their daily calorie intake in the form of carbohydrates, 35% in the form of fat, and 15% in the form of protein. There was no significant difference in IMF proportions between participants in the high-protein, low-carbohydrate diet relative to the control group.

### Quantitative Analysis

Studies were divided into three categories as follows: high-fat versus a control diet, high-carbohydrate/added carbohydrate diet versus a control diet, and low-calorie/starvation diet versus a control diet. Only studies that assessed changes in IMF proportions after the ingestion of high-fat diets were eligible for analysis. The number of studies in the other categories was limited and would bias the results of a meta-analysis since a small number of studies can result in a poor estimate of a distribution’s width and intervention effects (41, 42). Six of the eight studies assessing the impact of high-fat diets on IMF were included in the meta-analysis. One of the excluded studies only reported difference scores in their results and could not be included. The second excluded study reported their findings in a different metric, IMF:water ratio, relative to the absolute arbitrary metric values reported by the remainder of the studies. One of the included studies reported IMF proportion changes in two muscles areas, and these findings were reported as two separate entries in the meta-analysis. The pooled SMD of the proportion of IMF ~% change after a high-fat diet was estimated.

**Figure 2:**
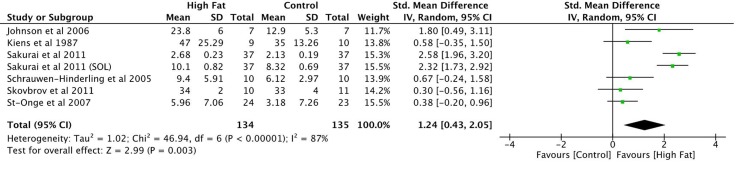
Meta-analysis on the effects of high-fat diets on IMTG proportions.

There were a total of 134 participants receiving a high-fat intervention and 135 people receiving a control intervention. SMDs were small for two of the seven reported observations and moderate for two other studies (Figure 2; overall SMD = 1.24, 95% CI 0.43–2.05) (9, 33, 36, 37). Two entries from Sakurai et al.’s (35) study presented large effect sizes. Johnson et al.’s (20) findings also presented a large SMD. All reported SMD values were in favor of increased IMF content in muscle after a high-fat diet intervention. The overall effect size was significant (*P* = 0.003; Figure 2). Heterogeneity in the sample was high (*I*^2^ = 87%). This can be attributed to both clinical and methodological differences between studies. Participant’s characteristics varied between studies. Three of the studies included samples consisting of physically trained men (9, 20, 35). Two of the remaining studies included healthy males with sedentary or moderate activity patterns (33, 37). Schrauwen-Hinderling et al. (36) did not state the physical activity patterns of their participants. Participants were all young to middle aged adults with ages varying between 18 and 50 years. The fat content of the diets and time period of the intervention within each of these studies varied as well. Finally, measurements were taken from three different muscles including the vastus lateralis muscle, soleus, and tibialis anterior using different measurement modalities across these studies.

### Sensitivity and Subgroup Analyses

The sensitivity analysis revealed results similar to the primary analysis results for the high-fat interventions studies (Table 3). Both results had significant effect sizes (sensitivity analysis SMD 1.49, *P* = 0.008). The subgroup analyses were not significant for percent males, trial type, measurement modality, and physical activity. However, significance was marginal for percent males (*P* = 0.05) and the measurement modality (*P* = 0.06) comparisons. There was a significant difference in the amount of IMF change between the types of muscle assessed after the dietary intervention. There were larger changes in the IMF of the tibialis anterior muscle relative to the vastus lateralis muscle after a high-fat diet intervention (9, 20, 35–39). Sakurai et al. (35) assessed changes at both the soleus and the tibialis anterior, and they found an increase in IMF in both muscle regions after a high-fat diet. The effect of IMF change in the soleus muscle and tibialis anterior muscle was comparable.

**Table 3:** Subgroup and sensitivity analysis.

Analysis	SMD (95% CI)	Significance P*
Primary analysis	1.24 (0.43, 2.05)	0.003
Sensitivity analysis: excluding studies with a quality value <20, smallest sample size <8, and those that do not specify participant characteristics	1.26 (0.23, 2.28)	0.02

**Subgroup analyses**		

**Percent males**		
Male only studies	1.4 (0.55, 2.25)	0.05
Mixed studies (21% M, 79% F)	0.38 (−0.20, 0.96)	
**Trial type**		
Controlled trial	0.58 (−0.30, 1.46)	0.23
Controlled crossover trial	1.35 (0.44, 2.25)	
**Measurement modality**		
H^1^MRS	1.55 (0.57, 2.54)	0.06
Biopsy	0.44 (−0.18, 1.05)	
**Muscle**		
Vastus lateralis	0.70 (0.17, 1.22)	Reference group
Soleus	1.35 (−0.55, 3.26)	0.52
Tibialis anterior	2.58 (1.96, 3.20)	<0.001*
**Physical activity**		
Active	1.09 (−0.09, 2.27)	0.26
Inactive	0.36 (−0.12, 0.84)	

*Subgroup analyses (Der Simonian and Laird) random-effects model*.

*CI, confidence interval; SMD, standardized mean difference*.

*Significance defined as P < 0.05.

## Discussion

Currently, there is an increasing focus on the importance of leading a healthy lifestyle, and diet is a central component to achieving this. Individuals, healthy or with illness, want to know what the ideal diet is for their physical health. A clinician cannot objectively prescribe a diet without knowing the individuals’ health profile. Muscle health is an essential component of wellness, as it regulates many physiological processes. Diet plays a dominant role in maintaining muscle health. Historically, the belief has been that high-fat and high-carbohydrate diets are harmful to health (14, 22). However, the effects of high-fat or high-carbohydrate diets on muscle composition, specifically IMF content, have not been described sufficiently in literature to provide a conclusive statement on the association between diet, IMF, and the potential health impacts.

Our systematic review suggests that high-fat diets, excessive caloric intake in the form of carbohydrates, and short-term starvation diets are associated with increases in IMF content. Hypocaloric diets did not result in any IMF proportion changes. Robust effects were found for increases in IMF content after high-fat diets. This effect was consistent across different patient populations with different proportions of fat in the diet. These findings are also in line with the evidence from the animal literature (12–14, 22). The subgroup analyses revealed more IMF storage after the dietary interventions in males relative to females, suggesting differences in male IMF metabolism or deposition relative to females. In addition, more IMF was found to be stored in the tibialis anterior muscle relative to the vastus lateralis, potentially due to its extensive use for walking and the need to store energy. Increases in the proportion of carbohydrates in the diet with decreased fat were shown to reduce IMF proportions (19, 20). This effect may have been due to decreases in diet fat content rather than increases in the proportion of carbohydrate consumption, since it was only present when the proportion of carbohydrates in the diet accounted for 75% of the participant’s caloric intake compared to 51%. Isocaloric diets with higher fat content present comparable effects to hypercaloric diets with added glucose and/or fructose consumption, suggesting that increases in IMF are both dependent on macronutrient intake and energy balance. Short-term starvation presented similar findings although it would be expected that non-caloric diets would result in decreases in IMF proportions as seen in animal models (18). It may be postulated that pathophysiological mechanisms associated with adapting to starvation alter the use of IMF reserves. This effect may also be attributed to the characteristics of the sample receiving this intervention. The two studies observing increased IMF stores after starvation had sample sizes composed of physically active men, whereas the study reporting no IMF change after starvation consisted of overweight men and women (19, 20). Increased physical activity may alter the utilization of IMF stores under starvation conditions. There is a need for additional studies to investigate the effects of diet on IMF further. The present review found one article assessing the impact of a high-protein diet on IMF, which found inconclusive results. However, a robust effect exists in the animal literature, suggesting that high-protein diets decrease IMF content and increase muscle mass (18, 19).

The impact on metabolic health associated with changes in IMF proportions in relation to different diets remains to be investigated further. There is consensus that high-fat diets and high-carbohydrate diets are associated with the development of disorders such as metabolic syndrome (43–45). IMF content has been shown to mediate this association with the additive effects of subcutaneous and visceral triglycerides (46). This study found an SMD of 1.24 on the change of IMF after the consumption of a high-fat diet. Studies by Mazzali et al. (47) and Janssen et al. (48) studied the effect of exercise/weight loss and diet/weight loss on IMF and insulin sensitivity, respectively. Mazzali et al. (47) reported a reduction in IMF proportions with an approximate effect size of 0.63, where participants’ insulin resistance significantly decreased. Janssen et al. (48) found that an energy-restrictive diet reduced IMF by an approximate effect size of 1.24. The insulin sensitivity of participants in this study increased. In light of this study’s findings, the increase of IMF (SMD 1.24) induced by high-fat diets may increase the risk for developing insulin resistance, and further evidence on this relationship is necessary. Although a reduction in the proportion of IMF has been reported with higher carbohydrate diets, these diets have also been associated with unhealthy blood lipid profiles (34); however, there is contrary evidence and further evidence is needed (49). Low-calorie, low-carbohydrate diets, which entail high-fat diet compositions and low caloric intake mitigate and can reverse elements of metabolic syndrome, namely insulin resistance, hypertension, poor lipid profile, and weight gain (50). It is unknown whether changes in IMF may mediate the health effects of this type of diet. The discrepancy between the health effects of isocaloric high-carbohydrate/high-fat diets and low-calorie, high-fat diets may be associated with the combined effect of low-calorie and low-carbohydrate intake, whereby it may tax the body into utilizing its fat reserves.

Due to the observed relationship between diet and IMF, we believe that it would be useful to assess IMF concentrations as a health outcome. Since IMF can reflect ones’ diet, imaging modalities such as ultrasound or H^1^MRS could be used to track the influence diet has on an individual and serve as an indication of health. For instance, increased IMF is found in elderly with poor gait (51). Based on the findings in animal models, a protein-rich, low-fat diet may be more suitable for the aging population to increase their muscle mass and decrease IMF stores (18, 19). Changes in muscle thickness and IMF can be monitored using feasible imaging modalities to assess their improvement. This outcome can also be generalized to clinical populations, such as those with metabolic syndrome, since high IMF is associated with insulin resistance. Or further, it can be used to gage how and where an individual is storing fat based on their diet, physical activity, training demands, and lifestyle. Measuring muscle thickness has been done clinically to monitor the effect of strengthening exercise on muscle thickness (52). Moreover, in view of the fact that diet influences the structure, composition, and metabolism of a muscle, clinical interventions and measurement techniques of the muscle will be affected by diet.

The subgroup analyses pointed to differences in IMF deposition between males and females, which are important to consider when imaging. Clinicians are taught to account for BMI when utilizing bedside imaging modalities such as ultrasound. Measurements acquired using imaging modalities will be influenced by BMI and diet-induced changes within anatomical structures—these two factors may have synergistic effects on image-based quantification of muscle characteristics. Different muscles also store fat differently, as demonstrated by the sensitivity analysis, and are influenced by physiology or lifestyle choices.

### Limitations

This is the first review assessing the effects of diet on IMF. The search for this review was comprehensive. Search terms covered IMF only, and three scientific and medical databases were used to identify articles discussing this topic in the literature. The risk of bias in the identified articles was low, lending to the strength of the conclusions drawn from the studies, with the exception of St-Onge et al.’s (33) study that presented high rates of attrition. The present review also presents some clinical and methodological variables that lend to the high heterogeneity observed among studies. The subgroup analyses indicated that the measurement modality, gender, and the type of muscle measured may have contributed to the heterogeneity observed. The studies examined in this review utilized various measurement modalities to characterize the proportion of IMF in its participants, namely H^1^MRS, and biopsy with transmission electron microscopy, extraction, or Oil Red staining. Most of the articles, 9 of the 13 chosen articles, utilized H^1^MRS when assessing the proportion of IMF in the muscle. In additionally, there was variability in the characteristics of participants in each study. Nine of the studies consisted of only men, of which five studies included physically active men. The remainder of the studies consisted of both men and women with various levels of physical activity. Seven of eight studies consuming high-fat diet interventions were male. Two of the four studies assessing the effects of increased carbohydrate consumption consisted of only males. Two of three studies assessing low-calorie/starvation diets consisted of males. Although there are between-study differences in IMF quantification methods and population characteristics, we do not believe they represent critical limitations to the interpretation or generalizability of the study’s results. Finally, since there were less than 10 studies in the meta-analysis assessing for IMF proportions following high-fat diets, we were unable to assess for publication bias among those studies both visually and statistically.

## Conclusion

A robust association between high-fat diets and IMF content is presented in this study. We suggest that the effect of diet on IMF content in humans be researched further, and the possible utility of IMF as a clinical marker of health also be investigated. There is a need for additional evidence on the single and combined effects of protein, carbohydrates, low-calorie, and starvation diets on IMF. With enough evidence, IMF may be a useful clinical tool to make specific dietary recommendations to combat chronic illness and improve health outcomes. The influence of low-calorie diets with low-carbohydrate proportions on IMF and metabolic health is a prime candidate for further investigation due to its promising effect on metabolic syndrome, type II diabetes, and obesity (50). Finally, it is unclear how diet influences the deposition and turnover of IMF as well as how this affects muscle metabolism and, in turn, systemic metabolic health. Further studies should assess the influence of diet on IMF proportion, and its association with the contractile properties and tensile strength of muscle as a marker of health.

## Author Contributions

All authors equally contributed to the collection, extraction, and analysis of data. In addition, equal efforts were contributed for the development of the final manuscript.

## Conflict of Interest Statement

The authors declare that the research was conducted in the absence of any commercial or financial relationships that could be construed as a potential conflict of interest.

## Appendix A

### Medline (Ovid) Search Strategy

   – – – – – – – – – – – – – – – – – – – – – – – – – – – – – – – – – – – – – – – –

1 intramyocellular triglyceride*1.tw,kw.
2 intra-myocellular triglyceride*1.tw,kw.
3 intramyocellular lipid*1.tw,kw.
4 intra-myocellular lipid*1.tw,kw.
5 intramyocellular fat*.tw,kw.
6 intra-myocellular fat*.tw,kw.
7 intramyocellular triacylglycerol*1.tw,kw.
8 intra-myocellular triacylglycerol*1.tw,kw.
9 intramuscular triglyceride*1.tw,kw.
10 intra-muscular triglyceride*1.tw,kw.
11 intramuscular lipid*1.tw,kw.
12 intra-muscular lipid*1.tw,kw.
13 intramuscular fat*1.tw,kw.
14 intra-muscular fat*1.tw,kw.
15 intramuscular triacylglycerol*1.tw,kw.
16 intra-muscular triacylglycerol*1.tw,kw.
17 intramuscular adipose tissue*.tw,kw.
18 intra-muscular adipose tissue*.tw,kw.
19 IMCL.tw,kw.
20 IMTG.tw,kw.
21 (skeletal muscle*1 adj2 (fat or triglyceride*1 or lipid*1 or triacylglycerol*1)).tw,kw.
22 or/1-21
23 22 not (exp animals/not exp humans/)
24 limit 23 to english language

   – – – – – – – – – – – – – – – – – – – – – – – – – – – – – – – – – – – – – – – –
